# How far is too far? Exploring the indications for robotic partial nephrectomy in a highly complex kidney tumor

**DOI:** 10.1590/S1677-5538.IBJU.2020.0059

**Published:** 2020-07-31

**Authors:** Andrea Minervini, Antonio Andrea Grosso, Fabrizio Di Maida, Andrea Mari, Gianni Vittori, Gianluca Muto, Marco Carini

**Affiliations:** 1 University of Florence Careggi Hospital Department of Oncologic, Minimally-Invasive Urology and Andrology Italy Department of Oncologic, Minimally-Invasive Urology and Andrology, Careggi Hospital, University of Florence, Italy

## Abstract

**Purpose:**

The conservative management of localized renal masses has been recently widened to cT2 tumors showing encouraging functional and oncological outcomes (1). This video aims to report the conservative management of a highly complex renal tumor treated with robotic pure enucleation in our center, specifically focusing on preoperative work-up, video-reported surgical steps and perioperative outcomes.

**Materials and Methods:**

A 63 year-old lady underwent CT scan revealing a single 75 x 68mm, mainly endophytic, right renal mass dislocating the vascular pedicle (cT3a). Two renal arteries and two veins were identified. PADUA, RENAL and simplified SPARE scores were 14a, 12a and 12 respectively. Since the contralateral kidney was hypotrophic, the indication for nephron-sparing approach was considered absolute. Preoperative surgical planning included the employment of 3D-virtual models (2).

**Results:**

Operative time was 150 minutes and warm ischemia time was 25 minutes. No major complication occurred. Histopathological analysis revealed a cromophobe renal cell carcinoma with extension to perirenal fat tissue (pT3a). Resection technique was classified as pure enucleation since Surface-Intermediate-Base (SIB) score was 0-0-0 (3, 4). At seven-months follow-up no signs of local or systemic recurrence were recorded. Postoperative CT-scan revealed optimal parenchymal volume preservation with last creatinine blood level of 1.16mg/dL.

**Conclusion:**

This video highlights how, in experienced hands, robotic partial nephrectomy represents a feasible, effective treatment option for surgical management of highly complex renal tumors. The employment of intraoperative ultrasonography and 3D-virtual models allowed to accurately tailor surgical approach, improving the perception of tumor anatomy and its vascularization and maximizing perioperative outcomes.
